# Dilated cardiomyopathy secondary to chronic cocaine abuse: a case report

**DOI:** 10.1186/1756-0500-6-536

**Published:** 2013-12-17

**Authors:** Chad J Cooper, Sarmad Said, Haider Alkhateeb, Emmanuel Rodriguez, Remi Trien, Shajeea Ajmal, Pedro A Blandon, German T Hernandez

**Affiliations:** 1 Department of Internal Medicine, Paul L. Foster School of Medicine, Texas Tech University Health Sciences Center at El Paso, El Paso, Texas, USA; 2 Division of Nephrology & Hypertension, Department of Internal Medicine, Paul L. Foster School of Medicine, Texas Tech University Health Sciences Center at El Paso, El Paso, Texas, USA

**Keywords:** Cocaine, Dilated cardiomyopathy, Congestive heart failure, Catecholamine

## Abstract

**Background:**

Cocaine is a potent sympathomimetic agent associated with the development of possible fatal cardiovascular complications. Dysrhythmias, acute myocardial infarction, hypertension and dilated cardiomyopathy are just some of many cardiovascular effects related to the abuse of cocaine.

**Case presentation:**

A 38-year-old Hispanic male with a past medical history of hypertension presented with a chief complaint of progressive shortness of breath. The patient confessed to the use of cocaine for almost 18 years once per week. On examination he was hypertensive and tachycardic with a systolic murmur over the 5th intercostal space at the level of the left mid-clavicular line. Laboratory workup revealed an elevated Brain natriuretic peptide; urine toxicology was positive for cocaine. 2D-echocardiogram showed dilated cardiomyopathy. Cardiac catheterization excluded angioischemic cause. He was managed medically and subsequently discharged with drug rehabilitation. On follow-up diagnostic evaluation after 5 months of cocaine cessation, his ejection function improved significantly.

**Conclusion:**

The exact incidence of cocaine related cardiomyopathy is unknown and likely underreported. The clinical course is abrupt and comparatively similar to other types of cardiomyopathy. The management is like other forms of cardiomyopathy; however β-blockers should be avoided. The myocardial dysfunction is reversible with abstaining from additional cocaine ingestion. Non-invasive testing should be performed after several months to re-evaluate the treatment response.

## Background

More than 14 million people worldwide, mostly within the age range of 15 to 64 years, consume cocaine. Men of 15–35 years represent the majority. Dose-dependent tachycardia, hypertension along with increased arousal is the first physiological response to cocaine use. Performance improvement, attentiveness, sense of positive self-image and euphoria often accompany the cumulative use of cocaine. End-organ-damage associated with cocaine can affect almost every organ system. In the United States cocaine, beside alcohol, is the most frequent cause of drug-associated emergency department (ED) visits. Cocaine results in a gradual addiction due to it vigorous sympathomimetic features with possible destructive cardiovascular effects. Cardiovascular complications account for a massive economic debt on the United States healthcare system. Dysrhythmias, acute myocardial infarction, myocarditis, hypertension, endocarditis, hypotensive shock, cerebral vascular accidents and dilated cardiomyopathy are several cardiovascular complications due to cocaine abuse [1].

Cocaine blocks the presynaptic dopamine and catecholamine uptake, resulting in post-synaptic sympathetic stimulation and dopaminergic receptor activation [2]. Peripheral vasoconstriction results in hypertension, tachycardia and an increase in afterload. Arrhythmias are likely to occur due to the altered autonomic action and cardiovascular resistance results in a decreased myocardial blood supply [3]. Negative inotropic events can also occur from cocaine abuse. Hypertrophy of the left ventricle and cardiomyopathy with significant reduction of the ejection fraction has been described in the setting of chronic cocaine consumption. Myocardial hypertrophy is likely to occur secondary to the temporary blood pressure elevation after cocaine use [4]. Smoking and alcohol use exacerbate the cardiotoxic impact of cocaine [5]. The unfavorable economic impact and awareness of the possible cardiovascular effects should be considered during the initial evaluation when young adults with heart failure symptomatology present for medical assessment.

## Case presentation

A 38-year-old Hispanic male presented with progressive dyspnea at rest since the morning, which became worse with exertion and lying flat. Similar episodes were also noticed approximately one month ago. During this time he was hospitalized for community-acquired-pneumonia and was discharged three days later. His other complaints included productive cough with whitish blood-tinged sputum, frontal headache, mild subjective fever and mid-sternal chest discomfort. Past medical history included only hypertension. No home medications were used. He confessed to regularly using cocaine once per week of various amounts for the last 18 years. His last cocaine use was on the night before admission. Other social history included occasional cigarette smoking, but no alcohol or intravenous drug use. He denied hazardous exposure.

**Figure 1:**
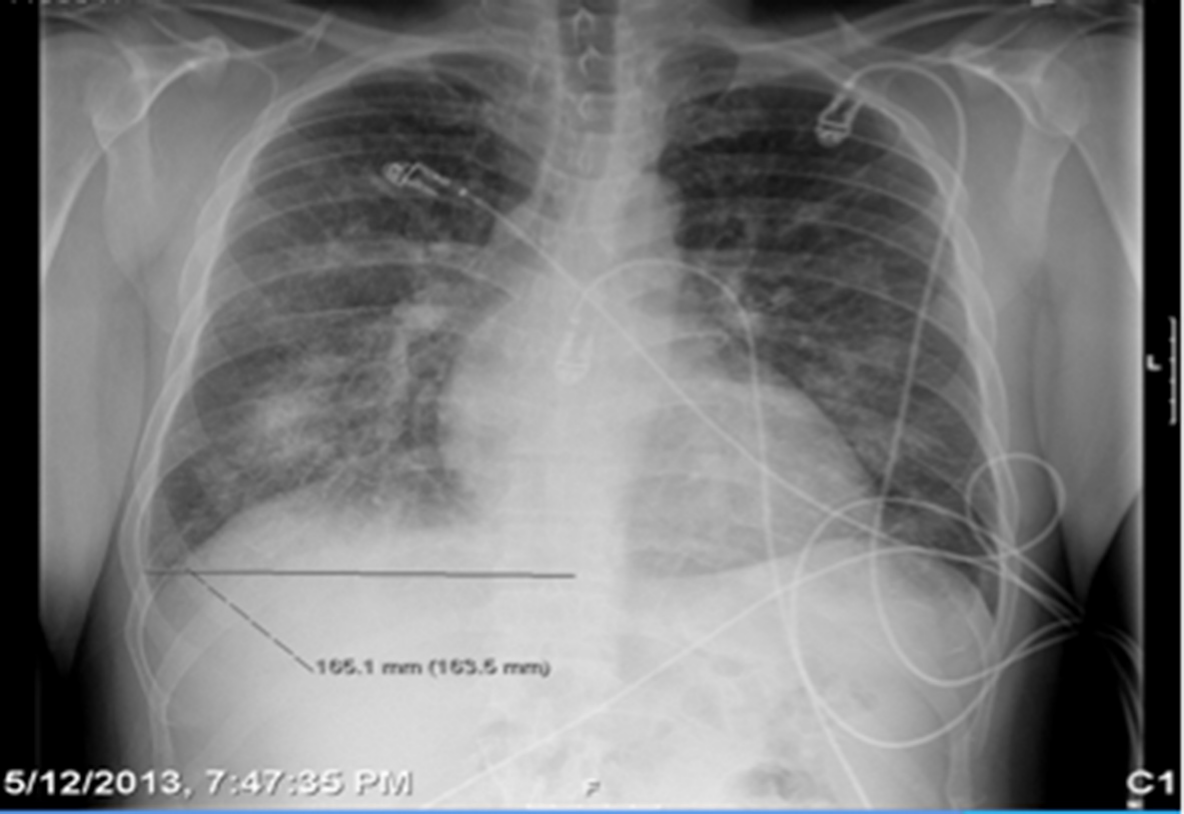
Chest X-ray- bilateral patchy opacities.

**Figure 2:**
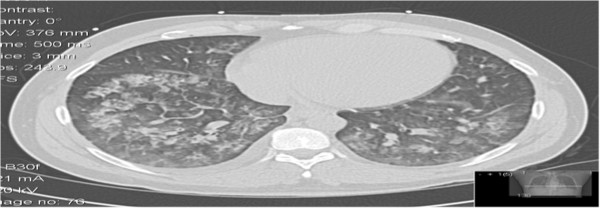
Computed tomography of the chest without contrast - bilateral peribronchovascular airspace consolidation, ground-glass opacity with interlobular septal thickening.

Initial vital signs revealed a tachycardic (heart rate: 104–110 beats per minute) and hypertensive (blood pressure: 179/121 mmHg) patient, with peripheral oxygen saturation of 86–89% on 2 L nasal cannula. Pertinent physical examination finding revealed multiple tattoos, mild nasal erythema without perforation signs of the nasal septum and a previous exploratory laparotomy scar (stab wound several years ago). Cardiovascular and lung examination were significant for jugular vein distention, tachycardia, 2/6 systolic murmur at mitral valve area with radiation to the left axilla and bilateral diffuse coarse crackles. Initial laboratory workup at admission was insignificant except for elevated brain nautriuretic peptide of 894 ng/L and positive urine toxicology screen for cocaine. Electrocardiogram on admission showed sinus tachycardia; left atrial enlargement, left ventricular hypertrophy and nonspecific T wave abnormalities over the anterior wall leads. Chest X-ray (Figure 1) showed bilateral patchy opacities representing pulmonary edema or possible hemorrhage. Computed tomography of chest (Figure 2) revealed bilateral peribronchovascular airspace consolidation, ground-glass opacity with interlobular septal thickening and small bilateral pleural effusions. Our differential included an etiology causing pulmonary edema versus a less likely pulmonary hemorrhage or atypical infection.

Due to his recent use of cocaine β-blockers were not used to control the hypertension however we opted to use amlodipine 10 mg daily instead. Pulmonary team was consulted; the impression was consistent with pulmonary edema from questionable congestive heart failure (CHF), which could have been from the history of cocaine use rather than a primary pulmonary cause. The recommendations included initiating a treatment with a diuretic agent, vasodilators and obtaining 2D-echocardiogram. Management consisted of amlodipine 10 mg daily, furosemide 20 mg twice daily, hydralazine 10 mg daily and lisinopril 10 mg daily. The 2D echocardiogram showed moderate left ventricle dilation (left ventricular end diastolic dimension LVEDD of 6.3 cm) without signs of apical ballooning; the left atrium with 4.9 cm moderate-severely dilated, moderate global hypokinesis of the left ventricle with an ejection fraction of 25%, stage 3 diastolic dysfunction and mild to moderate mitral regurgitation (Additional files 1, 2, 3, 4, 5 and 6). By the 4th day he was clinically asymptomatic and the pulmonary examination was unremarkable. Repeat chest x-ray showed almost complete resolution of bilateral patchy airspace consolidation phenomena however with some bibasilar persistence. The cardiology team was consulted for further recommendations, cardiac catheterization and the possibility of implantable cardioverter defibrillator (ICD) placement. The patient was taken for cardiac catheterization on hospital day 5 and ischemic cardiomyopathy was ruled out. An association between his symptoms and the binge consuming of cocaine was established. Cardiac biopsy was not recommended at this time. Re-evaluation of all the above findings including reconsidering a cardiac biopsy 5 months after the discharge was arranged. On the 6th day, the psychiatry service counseled the patient; he agreed to undergo a rehabilitation course in an inpatient institution. On the following day he was transferred to the rehabilitation center on his CHF medications.

Five months later and after successful completing of the treatment course at the rehabilitation center the patient was re-evaluated. Repeat echocardiogram was performed and showed an improved ejection fraction of 50-55%, improvement of the previously dilated size of the left ventricle (LVEDD from 6.3 cm to 5.4 cm) and mild-moderately dilated left atrium of 4.5 cm. The clinical condition was remarkably better, echocardiographic geometric changes partially resolved. Beta-blocker was added; cardiac biopsy was not done. Evidences for the strong association between previous cocaine abuse and newly diagnosed CHF were clinically confirmed.

## Discussion

The precise incidence of cocaine induced cardiomyopathy remains mysterious and probably underreported. The mechanism leading to develop cocaine-induced cardiomyopathy is not completely understood, however development of a coronary thrombus, increased oxidative stress, calcium flux sympathomimetic effects are contributing factors in its pathophysiologic formation [6]. The duration and quantity of cocaine use required to develop a subsequent cardiomyopathy is presently not clear. Cocaine induced cardiomyopathy occurs unexpectedly and without any significant prodrome [7]. Clinical manifestations are similar to other types of dilated cardiomyopathy. Presentation symptomatology can be nausea, dizziness, anxiety, dyspnea and palpitations. Only a few cases have been reported in the literature that describes a reversal of the cardiomyopathy after cocaine cessation in a young male that chronically used cocaine [8]. As described in our case, cocaine induced cardiomyopathy should always be considered when young male patients present with signs of left ventricular dysfunction, adrenergic excess or heart failure.

Typical echocardiographic features include a high left ventricular mass index, increased left ventricular mass and left ventricular dysfunction [8]. However it is difficult based on only the echocardiogram to distinguish it from other types of cardiomyopathy. The therapy of cocaine-induced cardiomyopathy is similar to the way that other types of cardiomyopathy are managed. Beta-blockers should not be considered initially; benzodiazepine is preferred to counteract the adrenergic effect. In the acute setting the addition of beta-blockers will adversely result in the alpha-adrenergic receptors being unopposed, therefore leading to coronary vasoconstriction, left ventricle wall stress and a hypertensive crisis [9]. As recommended in other types of cardiomyopathy, pharmacological agents such as, diuretics Angiotensin-Converting-Enzyme Inhibitors, Angiotensin-Receptor Blocker, vasodilators, or digoxin should be initially included.

It is strongly urged to consider a cardiology consultation during the hospital stay. By abstaining from cocaine use, the possibility of a favorable myocardial function reversibility is likely. However no functional improvement is expected if permanent myocardial injuries from prior infarctions occurred [10]. Once the patient is clinically stabilized, hospitalization for detoxification, psychiatric management to evaluate the need for pharmacotherapy, psychotherapy or counselling are highly recommended. Furthermore, it is suggested that repeat noninvasive testing to be performed after several months, preferably 4–8 months, to evaluate the myocardial functional response to medical therapy and cocaine cessation.

## Conclusion

Cocaine use should be considered if young patients presented with heart failure, mainly without other underlying risk factors. It is important to counsel these patients regarding the deleterious effects of cocaine abuse and the potential reversal of cardiac dysfunction with abstaining from the cocaine use.

## Consent

“Written informed consent was obtained from the patient for publication of this case report and accompanying images. A copy of the written consent is available for review by the Editor-in-Chief of this journal”.

## Competing interests

The authors declare that they have no competing interests.

## Authors’ contributions

CC was the primary physician in the team, who took care of the patient and arranged all required medical steps. He wrote the draft of the manuscript. SS was on the physician team as well. He participated in literature search, editing of the manuscript, rewriting, and final approval. HA was the main physician who collected literature and reviewed the final version of the manuscript. ER took care of the patient as an outpatient and contributed in updating the team about the patient`s condition. He also reviewed the final version, edited it and approved. RT analysed the manuscript and performed independent proofreading. SA corrected the last version of the manuscript and performed independent proofreading. PB is a senior physician who analysed the manuscript and performed an independent proofreading. GH is the senior physician of the team who controlled all the above-mentioned steps and made the final medical decisions. All authors read and approved the final manuscript.

## Supplementary Material

Additional file 1 Left parasternal long axis view demonstrating basal anteroseptal and inferolateral hypokinesis. Click here for file

Additional file 2 Left parasternal short axis view at the basal level demonstrating global reduced ejection fraction at this level. Click here for file

Additional file 3 Left ventricle apical four-chamber view representing a diffuse severely reduced ejection fraction. Click here for file

Additional file 4 Color-doppler of the left ventricle apical four-chamber view showing he mild-moderate mitral regurgitation. Click here for file

Additional file 5 Left ventricle five-chamber view demonstrating global reduced ejection fraction. Click here for file

Additional file 6 Left ventricle two-chamber view representing again the diffuse severely reduced ejection fraction. Click here for file
